# Genetic Mechanisms in Aspirin-Exacerbated Respiratory Disease

**DOI:** 10.1155/2012/794890

**Published:** 2011-08-07

**Authors:** Nami Shrestha Palikhe, Seung-Hyun Kim, Hyun Jung Jin, Eui-Kyung Hwang, Young Hee Nam, Hae-Sim Park

**Affiliations:** Department of Allergy and Clinical Immunology, Ajou University School of Medicine, San-5, Woncheondong, Youngtonggu, Suwon 442-721, Republic of Korea

## Abstract

Aspirin-exacerbated respiratory disease (AERD) refers to the development of bronchoconstriction in asthmatics following the exposure to aspirin or other nonsteroidal anti-inflammatory drugs. The key pathogenic mechanisms associated with AERD are the overproduction of cysteinyl leukotrienes (CysLTs) and increased CysLTR1 expression in the airway mucosa and decreased lipoxin and PGE2 synthesis. Genetic studies have suggested a role for variability of genes in disease susceptibility and the response to medication. Potential genetic biomarkers contributing to the AERD phenotype include *HLA-DPB1, LTC4S, ALOX5, CYSLT, PGE2, TBXA2R, TBX21, MS4A2, IL10, ACE, IL13, KIF3A, SLC22A2, CEP68, PTGER,* and *CRTH2* and a four-locus SNP set composed of *B2ADR, CCR3, CysLTR1*, and *FCER1B*. Future areas of investigation need to focus on comprehensive approaches to identifying biomarkers for early diagnosis.

## 1. Introduction

Aspirin-exacerbated respiratory disease (AERD) refers to the development of bronchoconstriction in asthmatics following the ingestion of aspirin or other nonsteroidal anti-inflammatory drugs. It is defined by a clinical syndrome associated with moderate-to-severe asthma and eosinophil inflammation in the upper and lower airways, resulting in chronic rhinosinusitis and asthma [1]. Additionally, the airways of AERD show epithelial disruption, cytokine production, and the upregulation of inflammatory molecules [2]. The prevalence of aspirin hypersensitivity in the general population ranges from 0.6 to 2.5% and is higher in asthmatics [3].

The dysregulation of arachidonic acid metabolism also accounts for the susceptibility to AERD. Metabolites involved are prostaglandins (PGs), leukotrienes (LTs), and thromboxane (TBX). Inhibition of COXs by acetyl salicylic acid (ASA) in the respiratory tract alters arachidonic acid metabolism, leading to a reduction in PGE2. This may increase AERD susceptibility by overproduction of CysLTs [4, 5]. The lipoxygenase (LOX) pathway produces the leukotrienes LTA4, LTB4, and LTC4 as metabolites. 15-lipoxygenase (15-LO) is one of the LOX family members and catalyses the conversion of arachidonic acid to 15-hydroxyperoxyeicosatetranoic acid (15-HPETE). 15-hydroxyeicosatetranoic (15-HETE), a more stable derivative of 15-HPETE, is another important product, which acts as an anti-inflammatory mediator and functional antagonist of LTs [6]. Further products of 15-HPETE include eoxins (EXs) EXA4 and 15-HETE can be conjugated with glutathione, leading to the formation of EXC4, EXD4, and EXE4. AERD has also been correlated with increased CysLT receptors: CysLTR1 and CysLTR2 [7–9]. The third CysLT receptor, the G protein-coupled receptor 17 (GPR17) [9], is located at an intermediate phylogenetic position between two distinct receptor families: the purinergic receptor (P2Y) and CysLT receptor for extracellular nucleotides and CysLTs, respectively, [10]. Overexpression of CysLTR1 was detected in the nasal mucosa of patients with AERD, compared with aspirin-tolerant asthma (ATA) [11]. Considering the pathogenic mechanism of AERD, various genetic markers have been suggested in various ethnic groups and are summarized in this paper.

## 2. Key Results Regarding Genetic Mechanisms

### 2.1. Leukotriene Related Genes and Their Mechanism

Based on evidence showing a close association of leukotrienes and AERD, initial research was performed on the association between *LTC4S* −444A  >  C promoter polymorphism and AERD. In the population investigated (Polish), the C allele was identified as a risk factor; however, this finding was not replicated in Japanese, American, or Korean populations [12–15]. SNPs of 5-lipoxygenase; *ALOX5* at −1708G  >  A, 21C  >  T, 270G  >  A, and 1728G  >  A and ALOX5 activating protein (*ALOX5AP*, 218A  >  G) were studied in a Korean population where it was discovered that the haplotype ALOX5 ht1 [G-C-G-A] was significantly higher in AERD than in ATA, suggesting a possible contribution of ALOX5 in AERD [16]. We identified three SNPs (−634C  >  T, −475A  >  C, and −336A  >  G) in the promoter region of CysLTR1, and mutant variants of these SNPs were associated with the AERD phenotype [17]. The mutant variants showed higher promoter activity, suggesting that these polymorphisms may modulate CysLTR1 expression increasing AERD susceptibility. In the case of *CysLTR2*, the frequencies of minor alleles for −819T  >  G, 2078C  >  T, and 2534A  >  G were significantly higher in the AERD group [18] when compared with ATA.

### 2.2. Cyclooxygenase, Prostanoid, and Human Leukocyte Antigen Markers and Related Mechanisms

It has been suggested that AERD is associated with both COX1 and COX2. Aspirin inhibits both of these proteins, with a greater effect on COX1. COX2 expression was downregulated in nasal polyps collected from AERD patients [19]. Decreased production of prostaglandin E2 (PGE2) by nasal epithelial cells of AERD has been observed [20]. PGE2 production in airway smooth muscle cells has been shown to downregulate COX2 mRNA expression [21]. Two SNPs of *TBXA2R*, −4684T  >  C, and +795T  >  C, were shown to be associated with the phenotype of AERD in a Korean population [22, 23]. The prostaglandin E2 receptor subtype 2 gene (*PTGER2*) was associated with the risk of AERD by decreasing the level of transcription, resulting in a reduction of the “PGE2 braking” mechanism of inflammation and involvement in the molecular mechanism underlying AERD in the Japanese population [24]. A further report in the Korean population showed that prostaglandin E2 receptor subtype 3 (*PTGER3*) may be an important genetic factor for aspirin intolerance in Korean asthmatics [25]. The human leukocyte antigen (HLA) allele *DPB*1*0301 was identified as a strong marker for AERD, because patients with this allele showed typical characteristics of AERD including a decreased forced expiratory volume in 1 s (FEV_1_) and increased prevalence of rhinosinusitis with nasal polyps [26], as previously noted in a Polish population [27].

### 2.3. Eosinophil-Related Genetic Mechanisms

Eosinophil infiltration into the upper and lower airways is a key feature of AERD. Increased numbers of eosinophils and mast cells have been observed in the bronchial mucosa of AERD [28, 29]. Recent studies demonstrated that the chemoattractant receptor molecule expressed in Th2 cells, the *CRTH2* −466T  >  C polymorphism, could increase serum and cellular eotaxin-2 production by lowering CRTH2 expression, leading to eosinophilic infiltration in AERD patients [30]. A further study indicated that the chemokine CC motif receptor (CCR3) may be related to eosinophil migration. The *CCR3* −520T  >  C was significantly associated with AERD patients where mRNA expression was also significantly increased after ASA provocation [31]. IL-13 polymorphisms at −1510A  >  C and 1055C  >  T are associated with the development of rhinosinusitis in AERD patients. *IL-13* Arg110Gln may be associated with an increased eosinophil count and eotaxin-1 level, leading to an increase in eosinophilic inflammation in the upper and lower airways of patients with AERD [32] (Table 1).

### 2.4. AERD and Viral Infection

Szczeklik has hypothesized that AERD develops as the result of chronic viral infection [33]. Viral respiratory infections have been suggested to contribute to allergic sensitization, leading to the development of asthma and in subjects with established asthma; they are known to exacerbate allergic disease [34]. Aspirin hypersensitivity is diminished in some AERD patients during acyclovir treatment of herpes simplex infection [35]. Moreover, elevated levels of IgG4, derived from chronic antigenic stimulation of viral origin, have been noted in AERD patients [36]. A further study investigating the exacerbation of AERD with airway infection of respiratory syncytial virus was reported [37]. Recently, a study indicated that the polymorphisms in the Toll-like receptor 3 (*TLR3*) gene, *TLR3* −299698G  >  T and 293391G  >  A, were associated with the AERD phenotype. TLR3 recognizes dsRNA, activates nuclear factors, and increases interferon-gamma, which is a signal to other cells and increases antiviral defenses. As functional deterioration of TLR3 can predispose individuals to increased susceptibility to viral infections, the detection of TLR3 polymorphisms may be informative for risk assessment in AERD susceptibility [38]. The suggested mechanism is that specific cytotoxic lymphocytes are produced in response to viral infection. Activity of these lymphocytes is suppressed by PGE2, which is produced by pulmonary alveolar macrophages. If PGE2 levels are decreased, cytotoxic reactions are preceded by COX inhibitors and cytotoxic lymphocyte-mediated attacks lead to the destruction of virus affected cells in the respiratory tract. Reactive oxygen species, toxic metabolites, and mediators released then precipitate asthma attacks.

### 2.5. Other Suggested Mechanisms

The ubiquitin-proteasome pathway-related gene (*UBE3C)* has been recently studied in a Korean population and indicated that rs3802122 and rs6979947 is associated with AERD [39]. A further study indicated that the kinesin family number 3A (*KIF3A*) gene and its polymorphism might have an effect on AERD, because rs3756775 revealed a significant association with the percentage decline in FEV_1_ after aspirin provocation [40]. Recently, the genome-wide methylation profile of nasal polyps showed that genes involved in lymphocyte proliferation, cell proliferation, leukocyte activation, cytokine biosynthesis, immune responses, inflammation, and immunoglobulin binding were hypomethylated. In the arachidonic pathways, PGDS, ALOX5AP, and LTB4R were hypomethylated whereas PTGES was hypermethylated [41]. The calcium channel voltage-dependent gamma subunit 6 (*CACNG6*) gene encodes a protein that stabilizes the calcium channel. *CACNG6* has been studied in AERD, which revealed that rs192808C  >  T may be associated with the risk of AERD in a Korean population [42].

### 2.6. AERD and Genome-Wide Studies

Genome-wide association studies (GWAS) have recently emerged as a technology that can predict genetic variations across the genome associated with human diseases and clinical responses to drug treatment. Recently, GWAS for asthma and related phenotypes have reported several susceptible genes. Candidate gene approaches have been used for most of the genetic association studies of AERD. GWAS suggested that the nonsynonymous *CEP68* rs 7572857G  >  A variant, replacing glycine with serine, showed a higher decline in FEV_1_ due to aspirin provocation than other variants and could be a susceptible gene for AERD. Gly74Ser could also affect the polarity of the protein structure [43].

### 2.7. Gene-Gene Interactions

Gene-gene interactions have also been proposed in the pathogenesis of AERD, and a few studies indicated that the genetic effects of CysLTs and *LTC4S* −444A  >  C synthesis increased the lower level of FEV_1_ after lysine ASA inhalation [18]. *TBXA2R* 795T  >  C polymorphism was associated with* HLA* *DPB*1*0301 in AERD patients compared with ATA [23]. Recently, a synergistic effect between the *TGF-beta1*-509C/T and *IL-10*-1082A/G polymorphisms on the phenotype of AERD was noted when stratified by the presence of rhinosinusitis [44]. Moreover, Kim et al. reported a significant epistatic effect with a four-locus genetic interaction in the susceptibility to aspirin intolerance in asthmatic patients. This model includes four SNPs: *B2ADR −*46A  >  G, *CCR3 −*520T  >  G, *CysLTR1 −*634C  >  T, and *FCER1B *−109T  >  C [45]. These findings should be validated further in other cohorts.

## 3. Conclusions

AERD often produces a moderate-to-severe phenotype; however, diagnosis in these patients is challenging despite the availability of various techniques. A hypothesis has been put forward, mostly focused on the overproduction of CysLTs and arachidonic acid pathways. Most of the genetic studies have been performed using techniques such as GWAS and the candidate gene approach. However, replication studies in different ethnic groups will be essential to validate the reported data and apply this knowledge in clinical practice. Future areas of investigation should focus on identification of biomarkers for early diagnosis with various diagnostic techniques. These genetic studies will be able to extend our understanding about the molecular genetic mechanism of AERD and to find a genetic marker for predicting drug responses or hypersensitivity reactions. Furthermore, this will be helpful for the determination of new diagnostic tools and therapeutic interventions.

## Figures and Tables

**Table 1:** Genetic mechanisms of AERD.

Gene name	SNPs	Clinical Phenotype	Mechanism
Leukotriene synthesis			
LTC4S	−444A > C	C allele had high genotype frequency compared with A allele	C allele may be the risk allele due to overproduction of CysLTs
ALOX5	−1708G > A, 21C > T, 270G > A, 1728G > A	ALOX5 ht1(GCGA) had higher haplotype frequency	ALOX5 ht1(GCGA) may be the risk haplotype
**CYSLTR1 **	**−634C > T, −475A > C, −336A > G**	ht2(TCG) showed higher frequency in AERD and higher promoter activity	Higher CysLTR1 mRNA expression may be responsible for pathogenesis
CYSLTR2	−819T > C	the frequencies of rare allele were increased in AERD and fall in FEV1 after aspirin provocation	Elevation of CysLTs production

COX/PG pathway and HLA allele			
PTGER	rs7543182 rs959	These two polymorphisms retained their susceptibility to aspirin intolerance in first and second cohorts	PTGER3 might play a significant role in aspirin hypersensitivity
TBXA2R	+795T > C	AERD patients with homozygous +795 C allele had a greater percent fall in FEV1 after aspirin exposure compared with TBXA2R+795 CT or TT genotypes.	TBXA2R+795T > C may increase bronchoconstrictive response to ASA
**HLA **	DPB1*0301	Patients with DPB1*0301 allele had higher prevalence of Rhino-sinusitis and lower FEV1 values.	HLA markers may be important for LTRA therapy
Gene name	SNPs	Clinical Phenotype	Mechanism

Eosinophil activation			
**CRTH2**	**−466T > C**	−466T allele had higher frequency in AERD and increased serum, cellular eotaxin-2 production and lower mRNA expression	−466T allele may be the risk allele by activation of eosinophils
**CCR3**	**−520T > C**	The frequencies of rare genotypes were higher in AERD and −520G allele showed higher promoter activity	Higher mRNA expression of CCR3 may cause eosinophil activation
IL 13	1510A > C, 1055C > T, Arg110Gln	Increase eotaxin-1 and peripheral eosinophil count	Eosinophil activation may occur

Mast cell activation			
**FCERIG**	** −237A > G −344C > T**	AA type of −237A > G showed high serum total IgE; CC/CT of −344C/T had higher SEA	Mast cells may be activated
MS4A2R	E237G	FcER1b −109T allele had higher frequency and high promoter activity	Increased mRNA expression of −109T allele may cause mast cell activation mediated by MS4A2R receptor

Other mechanisms			
IL-10 and TGF-*β*1	−1082 A > G and −509C > T	The frequency of rare alleles (the CT or TT genotype of TGF-*β*1) 509C/T and AG or GG genotype of (IL-10 )1082A/G was significantly higher in AERD and −1082G had higher promoter activity	Alteration in IL-10 production caused by the −1082A/G in IL-10 may contribute to disease pathogenesis which is strengthened by a genetic interaction with TGF-*β*1.
ACE	−262A > T, −115T > C	The frequencies of the rare alleles were higher in AERD −262T had lower promoter activity and fall of FEV1 after aspirin provocation	Downregulation of ACE expression
KIF3A	rs 3756775	Fall of FEV1 and higher mRNA expression of KIF3A in the ASA induced bronchial epithelial cells and protein expression in nasal polyp epithelia in AERD	Abnormality of cilia predisposing to AERD
SLC6A12	rs499368, rs557881	The minor allele frequencies were higher in AERD and fall of FEV1 after aspirin provocation	GABA signaling pathway in the airway epithelium may play a role
CEP68	7572857G > A	Fall of FEV1 after aspirin provocation by A allele	Change in polarity of the protein structure due to nonsynonymous SNP which replaces Gly with Ser

IL13: interleukin 13, CCR3: chemokine receptor 3, CRTH2: chemoattractant receptor, IL10: interleukin 10, TGF: transforming growth factor, MS4A2R: high affinity immunoglobulin epsilon receptor beta-subunit (FcERI) TBXA2R: thromboxane receptor, CysLTR1: cysteinyl leukotriene 1, CysLTR2: cysteinyl leukotriene 2, ALOX5: arachidonate 5 lipoxygenase, HLA: human leukocyte antigen, LTC4S: leukotriene C4, ACE: angiotensin-converting enzyme KIF3A: kinesin family number 2A, SLC22A2: solute carrier family 6, CEP68: centrosomal protein, PTGER: prostanoid gene, TEC: total eosinophilic count, TF: transcription factor, MAZ: myc-associated zinc finger protein, SEA: Staphylococcus enterotoxin A, FEV_1_: forced expiratory volume in 1 s, AERD: aspirin-exacerbated respiratory disease.

## References

[1] Szczeklik A, Nizankowska E, Duplaga M (2000). Natural history of aspirin-induced asthma. AIANE Investigators. European Network on Aspirin-Induced Asthma. *European Respiratory Journal*.

[2] Szczeklik A, Stevenson DD (2003). Aspirin-induced asthma: advances in pathogenesis, diagnosis, and management. *Journal of Allergy and Clinical Immunology*.

[3] Palikhe NS, Kim JH, Park HS (2009). Update on recent advances in the management of aspirin exacerbated respiratory disease. *Yonsei Medical Journal*.

[4] Picado C (2002). Aspirin-intolerant asthma: role of cyclo-oxygenase enzymes. *Allergy*.

[5] Çelik G, Bavbek S, Misirligil Z, Melli M (2001). Release of cysteinyl leukotrienes with aspirin stimulation and the effect of prostaglandin E2 on this release from peripheral blood leucocytes in aspirin-induced asthmatic patients. *Clinical and Experimental Allergy*.

[6] Chavis C, Vachier I, Godard P, Bousquet J, Chanez P (2000). Lipoxins and other arachidonate derived mediators in bronchial asthma. *Thorax*.

[7] Hui Y, Funk CD (2002). Cysteinyl leukotriene receptors. *Biochemical Pharmacology*.

[8] Capra V (2004). Molecular and functional aspects of human cysteinyl leukotriene receptors. *Pharmacological Research*.

[9] Ciana P, Fumagalli M, Trincavelli ML (2006). The orphan receptor GPR17 identified as a new dual uracil nucleotides/cysteinyl-leukotrienes receptor. *EMBO Journal*.

[10] Parravicini C, Ranghino G, Abbracchio MP, Fantucci P (2008). GPR17: molecular modeling and dynamics studies of the 3-D structure and purinergic ligand binding features in comparison with P2Y receptors. *BMC Bioinformatics*.

[11] Sousa AR, Parikh A, Scadding G, Corrigan CJ, Lee TH (2002). Leukotriene-receptor expression on nasal mucosal inflammatory cells in aspirin-sensitive rhinosinusitis. *New England Journal of Medicine*.

[12] Sanak M, Pierzchalska M, Bazan-Socha S, Szczeklik A (2000). Enhanced expression of the leukotriene C4 synthase due to overactive transcription of an allelic variant associated with aspirin-intolerant asthma. *American Journal of Respiratory Cell and Molecular Biology*.

[13] Kawagishi Y, Mita H, Taniguchi M (2002). Leukotriene C4 synthase promoter polymorphism in Japanese patients with aspirin-induced asthma. *Journal of Allergy and Clinical Immunology*.

[14] Van Sambeek R, Stevenson DD, Baldasaro M (2000). 5’ Flanking region polymorphism of the gene encoding leukotriene C4 synthase does not correlate with the aspirin-intolerant asthma phenotype in the United States. *Journal of Allergy and Clinical Immunology*.

[15] Choi JH, Kim SH, Bae JS (2005). Lack of an association between a newly identified promoter polymorphism (−1702G > A) of the leukotriene C4 synthase gene and aspirin-intolerant asthma in a Korean population. *Tohoku Journal of Experimental Medicine*.

[16] Choi JH, Park HS, Oh HB (2004). Leukotriene-related gene polymorphisms in ASA-intolerant asthma: an association with a haplotype of 5-lipoxygenase. *Human Genetics*.

[17] Kim SH, Oh JM, Kim YS (2006). Cysteinyl leukotriene receptor 1 promoter polymorphism is associated with aspirin-intolerant asthma in males. *Clinical and Experimental Allergy*.

[18] Park JS, Chang HS, Park CS (2005). Association analysis of cysteinyl-leukotriene receptor 2 (CYSLTR2) polymorphisms with aspirin intolerance in asthmatics. *Pharmacogenetics and Genomics*.

[19] Picado C, Fernandez-Morata JC, Juan M (1999). Cyclooxygenase-2 mRNA is downexpressed in nasal polyps from aspirin- sensitive asthmatics. *American Journal of Respiratory and Critical Care Medicine*.

[20] Kowalski ML, Pawliczak R, Wozniak J (2000). Differential metabolism of arachidonic acid in nasal polyp epithelial cells cultured from aspirin-sensitive and aspirin-tolerant patients. *American Journal of Respiratory and Critical Care Medicine*.

[21] Chambers LS, Black JL, Ge Q (2003). PAR-2 activation, PGE2, and COX-2 in human asthmatic and nonasthmatic airway smooth muscle cells. *American Journal of Physiology—Lung Cellular and Molecular Physiology*.

[22] Kim SH, Kim YK, Park HW (2007). Association between polymorphisms in prostanoid receptor genes and aspirin-intolerant asthma. *Pharmacogenetics and Genomics*.

[23] Kim SH, Choi JH, Park HS (2005). Association of thromboxane A2 receptor gene polymorphism with the phenotype of acetyl salicylic acid-intolerant asthma. *Clinical and Experimental Allergy*.

[24] Jinnai N, Sakagami T, Sekigawa T (2004). Polymorphisms in the prostaglandin E2 receptor subtype 2 gene confer susceptibility to aspirin-intolerant asthma: a candidate gene approach. *Human Molecular Genetics*.

[25] Park BL, Park SM, Park JS (2010). Association of PTGER gene family polymorphisms with aspirin intolerant asthma in Korean asthmatics. *BMB Reports*.

[26] Choi JH, Lee KW, Oh HB (2004). HLa association in aspirin-intolerant asthma: DPB1*0301 as a strong marker in a Korean population. *Journal of Allergy and Clinical Immunology*.

[27] Dekker JW, Nizankowska E, Schmitz-Schumann M (1997). Aspirin-induced asthma and HLA-DRB1 and HLA-DPB1 genotypes. *Clinical and Experimental Allergy*.

[28] Nasser S, Christie PE, Pfister R (1996). Effect of endobronchial aspirin challenge on inflammatory cells in bronchial biopsy samples from aspirin-sensitive asthmatic subjects. *Thorax*.

[29] Nasser SMS, Pfister R, Christie PE (1996). Inflammatory cell populations in bronchial biopsies from aspirin-sensitive asthmatic subjects. *American Journal of Respiratory and Critical Care Medicine*.

[30] Palikhe NS, Kim SH, Cho BY, Ye YM, Choi GS, Park HS (2010). Genetic variability in CRTH2 polymorphism increases eotaxin-2 levels in patients with aspirin exacerbated respiratory disease. *Allergy*.

[31] Kim SH, Yang EM, Lee HN, Choi GS, Ye YM, Park HS (2010). Association of the CCR3 gene polymorphism with aspirin exacerbated respiratory disease. *Respiratory Medicine*.

[32] Palikhe NS, Kim SH, Cho BY (2010). IL-13 gene polymorphisms are associated with rhinosinusitis and eosinophilic inflammation in aspirin intolerant asthma. *Allergy, Asthma and Immunology Research*.

[33] Szczeklik A (1988). Aspirin-induced asthma as a viral disease. *Clinical Allergy*.

[34] Van Rijt LS, Geurts Van Kessel CH, Boogaard I, Lambrecht BN (2005). Respiratory viral infections and asthma pathogenesis: a critical role for dendritic cells?. *Journal of Clinical Virology*.

[35] Yoshida S, Sakamoto H, Yamawaki Y (1998). Effect of acyclovir on bronchoconstriction and urinary leukotriene E4 excretion in aspirin-induced asthma. *Journal of Allergy and Clinical Immunology*.

[36] Szczeklik A, Schmitz-Schumann M, Nizankowska E, Milewski M, Roehlig F, Virchow C (1992). Altered distribution of IgG subclasses in aspirin-induced asthma: high IgG4, low IgG1. *Clinical and Experimental Allergy*.

[37] Filipowicz E, Sanak M (2003). Exacerbation of aspirin-induced asthma associated with RSV infection. *Przeglad lekarski*.

[38] Palikhe NS, Kim S-H, Kim J-H, Losol P, Ye Y-M, Park H-S (2011). Role of Toll-like receptor 3 variants in aspirin-exacerbated respiratory disease. *Allergy, Asthma and Immunology Research*.

[39] Lee JS, Kim JH, Bae JS (2010). Association analysis of UBE3C polymorphisms in Korean aspirin-intolerant asthmatic patients. *Annals of Allergy, Asthma and Immunology*.

[40] Kim JH, Cha JY, Cheong HS (2010). KIF3A, a Cilia Structural Gene on Chromosome 5q31, and Its Polymorphisms Show an Association with Aspirin Hypersensitivity in Asthma. *Journal of Clinical Immunology*.

[41] Cheong HS, Park SM, Kim MO (2011). Genome-wide methylation profile of nasal polyps: relation to aspirin hypersensitivity in asthmatics. *Allergy*.

[42] Lee JS, Kim JH, Bae JS (2010). Association of CACNG6 polymorphisms with aspirin-intolerance asthmatics in a Korean population. *BMC Medical Genetics*.

[43] Kim JH, Park BL, Cheong HS (2010). Genome-wide and follow-up studies identify CEP68 gene variants associated with risk of aspirin-intolerant asthma. *PLoS ONE*.

[44] Kim SH, Yang EM, Lee HN, Cho BY, Ye YM, Park HS (2009). Combined effect of IL-10 and TGF-*β*1 promoter polymorphisms as a risk factor for aspirin-intolerant asthma and rhinosinusitis. *Allergy*.

[45] Kim SH, Jeong HH, Cho BY (2008). Association of four-locus gene interaction with aspirin-intolerant asthma in Korean asthmatics. *Journal of Clinical Immunology*.

